# HIV cascade of care in Greece: Useful insights from additional stages

**DOI:** 10.1371/journal.pone.0207355

**Published:** 2018-11-15

**Authors:** Georgia Vourli, Georgios Nikolopoulos, Vasilios Paparizos, Athanasios Skoutelis, Symeon Metallidis, Panagiotis Gargalianos, Antonios Papadopoulos, Maria Chini, Nikolaos V. Sipsas, Mina Psychogiou, Georgios Chrysos, Helen Sambatakou, Charalambos Gogos, Olga Katsarou, Dimitra Paraskeva, Nikos Dedes, Giota Touloumi, on behalf of the Greek HIV Prevention Group

**Affiliations:** 1 Dept of Hygiene, Epidemiology and Medical Statistics, Medical School, National and Kapodistrian University of Athens, Athens, Greece; 2 Medical School, University of Cyprus, Nicosia, Cyprus; 3 AIDS Unit, Clinic of Venereologic & Dermatologic Diseases, Athens University, Medical School, Syngros Hospital, Athens, Greece; 4 Infectious Diseases & HIV Division, Dept of Internal Medicine, Evangelismos Athens General Hospital, Athens, Greece; 5 Aristotle University HIV Unit, AHEPA University Hospital, Thessaloniki, Greece; 6 Infectious Diseases Unit, 1^st^ Dept of Medicine, "G. Gennimatas" Athens General Hospital, Athens, Greece; 7 4th Dept of Internal Medicine, Athens Medical School, Attikon University Hospital, Athens, Greece; 8 Infectious Diseases Unit, Red Cross General Hospital of Athens, Athens, Greece; 9 Pathophysiology Department, “Laikon” General Hospital, Medical School, National and Kapodistrian University of Athens, Athens, Greece; 10 “Laikon” Athens General Hospital and Athens University, Medical School, Athens, Greece; 11 Infectious Disease Unit, “Tzaneio” General Hospital of Piraeus, Piraeus, Greece; 12 Infectious Disease Unit, 2^nd^ Dpt. of Internal Medicine, Athens University, Medical School, Hippokration General Hospital, Athens, Greece; 13 Infectious Diseases Section, 1^st^ Dept of Internal Medicine, Patras University Hospital, Patras, Greece; 14 Blood Transfusion Unit, and National Reference Centre for Congenital Bleeding Disorders, Laikon General Hospital, Athens, Greece; 15 HIV office, Hellenic Center of Disease Control and Prevention, Athens, Greece; 16 Positive voice, Non-Governmental Organization, Athens, Greece; New York Blood Center, UNITED STATES

## Abstract

**Background:**

Aiming to eliminate HIV infection, UNAIDS has set a global “90-90-90” target by 2020. We sought to construct a 6-stages HIV Cascade of Care (CoC) in Greece, overall and by risk group, to assess risk-group and stage-specific progress in achieving the UNAIDS target.

**Patients and methods:**

Combining data from the HIV/AIDS surveillance system and a population-based HIV cohort study, the CoC included: i) number of people living with HIV (PLHIV) by end of 2013; ii) proportion of PLHIV ever diagnosed; iii) proportion of diagnosed linked-to-care iv) proportion of linked-to-care ever initiating antiretroviral therapy (ART); v) proportion of treated who retained-in-care vi) proportion of those retained-in-care who were virally suppressed (≤200 copies/mL) at their last visit (01/07/2012-31/12/2013).

**Results:**

In 2013, 14147 PLHIV were in Greece. Overall, proportions of each stage in the cascade were: 78.4% diagnosed; 86% linked-to-care; 78.5% initiated ART; 86.4% retained-in-care, and 87.1% virally suppressed. Totally, 42.6% of all PLHIV were virally suppressed. The percentage diagnosed was lower among heterosexual men and women (heterosexuals) than in MSM (men who have sex with men) or PWID (people who inject drugs). Most MSM were linked to care (97.2% of diagnosed) while a substantial proportion of PWID were not (80.8% of diagnosed). Once treated, PWID remained in care in similar proportions to MSM. Unlike PWID, a high proportion of the retained in care MSM and heterosexuals achieved viral suppression.

**Conclusions:**

At the end of 2013, we identified gaps in the HIV CoC in Greece, which differed across risk groups. Targeted interventions are critical in optimizing early diagnosis and timely linkage. A 6-stage CoC, stratified by risk group, can inform strategic public health planning in improving HIV treatment outcomes.

## Introduction

Combined antiretroviral therapy (ART) improves both individual patient survival and prevention of onward HIV transmission [1, 2]. However, despite advances in treatment and innovations in prevention, such as pre-exposure prophylaxis (PrEP), HIV remains a major global health issue. Approximately 1.8 million people acquired HIV in 2017, indicating a slower than expected HIV incidence decline [3]. Given the high effectiveness of ART, HIV transmission and associated mortality are largely attributed to the substantial number of HIV-infected individuals who remain undiagnosed and/or untreated. Thus, it is now well recognized that the HIV response should go beyond the availability of ART; it includes timely diagnosis, linkage to and retention in care [4]. Unless a sufficient number of the people living with HIV (PLHIV) are engaged in all the above-mentioned steps, the public health of ART provision cannot be realized.

Aiming to eliminate HIV globally and taking into account a holistic approach to HIV care, UNAIDS has set the 90-90-90 target by 2020: 90% of all PLHIV to be diagnosed; 90% of those diagnosed to be on antiretroviral treatment (ART); and 90% of those on ART to become virally suppressed [5]. Many countries have already created national HIV Cascades of Care (CoC). In accordance with the UNAIDS 90-90-90 target, the usual CoC consists of 4 sequential stages: a) the number of PLHIV, b) the number or proportion of PLHIV who have received an HIV diagnosis, d) have received treatment, and d) have achieved viral suppression. Although some high-income countries have achieved the targets in one or two of these stages, most countries are not on track to achieve the overall target by 2020. The percentage of virally suppressed individuals among PLHIV ranges between 52% and 59% in Western European countries, but it is lower in the United States of America (around 30%) and in Eastern European countries (around 20%) [6–8]. Sweden and Denmark are the first two countries to achieve the 90-90-90 goal [9–11].

While a 4-stage CoC provides useful public health information, additional stages, such as the proportion of the diagnosed people who are linked to care and the proportion of those who initiated ART are retained in care, could provide critical insight to optimize treatment responses at the national level. Furthermore, the construction of a 6-stage CoC stratified by HIV risk group could help to identify specific gaps in each risk group and, thus, to develop and implement targeted interventions. Nonetheless, 6-stage CoCs, both for the overall population of PLHIV and by risk group are less often constructed, mainly due to lack of relevant data.

The aim of this work was to characterize a 6-stage Greek CoC, overall and by risk group, as to identify stage-specific gaps. For that, we combined information from both the HIV surveillance system and the Greek cohort of HIV-infected individuals, Athens Multicenter AIDS Cohort Study (AMACS).

## Methods

The CoC was constructed combining information from the HIV/AIDS surveillance system operated by the Hellenic Center for Disease Control and Prevention **(**HCDCP) and from the AMACS. The HIV/AIDS surveillance system started operating when the first AIDS cases were diagnosed in Greece in the early 1980s. All HIV clinics and laboratories nationwide are required to report HIV and AIDS cases, and deaths to HCDCP. Reporting data on treatment initiation and changes are also mandatory. AMACS is a nationwide cohort that began in 1996 and included all HIV-infected individuals followed in specific collaborating HIV clinics. Currently, the largest 13 out of 18 HIV specialized treatment clinics in Greece participate in AMACS. In accordance with data protection national policy, data are provided by the clinics fully anonymized to the cohort investigators. AMACS has been approved by the National and Kapodistrian University of Athens Ethics Committee and the National Organization for Medicines (E.O.F.). Six-stage CoCs were constructed for 2013, the most recent year with available data, for the entire HIV-infected population in the country, by HIV risk group [men having sex with men (MSM), heterosexuals, people who inject drugs (PWID)] and for migrants.

### Definition of stages

#### Stage 1: Number of PLHIV

Stage 1 was defined as the estimated number of PLHIV, diagnosed and undiagnosed, who were alive and living in Greece by the end of 2013. Thus, people known to be dead or known to have emigrated were excluded. Deaths are reported to the HCDCP, but migration data are not routinely collected. The number of PLHIV was estimated by applying the Incidence Method of the European Center for Disease Control (ECDC) HIV modelling tool on surveillance data collected at the HCDCP [12]. Datasets used as inputs for the ECDC tool were prepared for the entire HIV-infected population and for important risk groups including MSM, which is the population disproportionately affected by HIV, PWID who experienced a serious outbreak between 2011 and 2014, and heterosexuals. Briefly, the Incidence Method incorporates a multi-state back-calculation mathematical model that estimates HIV incidence and the size of the undiagnosed population, using data on reported HIV and AIDS cases, and information on CD4 count at the time of diagnosis [13]. Based on this method, model parameters for the number of HIV infections were initially unknown and were estimated by comparing model outcomes with observed data. After this procedure, the numbers of PLHIV, both overall and for the specific subgroups mentioned above, were estimated and 95% confidence intervals (CI) were calculated using bootstrapping techniques.

#### Stage 2: Proportion of PLHIV who are diagnosed

Stage 2 was defined as the proportion of PLHIV who were diagnosed with HIV. The number of diagnosed individuals was retrieved from the HIV/AIDS registry at the HCDCP. Duplicates were checked through matching records by birth date, sex, and name/surname initials; missing information in key variables (e.g. risk group) were in most cases retrieved through regularly updated information. Data on migrant status (country of origin) was also collected by HCDCP. The number of diagnosed individuals was divided by the upper and lower confidence limits of the estimated PLHIV to reflect the uncertainty in the estimate derived in stage 1.

#### Stage 3: Proportion of the diagnosed who were linked to care

Stage 3 was defined as the proportion of those diagnosed who were linked-to-care. This was estimated based on HCDCP data; Individuals with at least one CD4 measurement reported, an AIDS diagnosis or those who initiated ART, were considered as linked to care. HIV clinics routinely report data on CD4 counts at diagnosis to the HCDCP since 2009. Data on CD4 counts at diagnosis before 2009 have been retrospectively collected although information was not always available. As a cross-check, the number of linked to care individuals was also estimated based on the number of AMACS participants, taking into account its coverage (i.e. accounting for the number of HIV-infected individuals followed in clinics not participating in AMACS).

#### Stage 4: Proportion initiating ART

Stage 4 was defined as the proportion of those linked-to-care who initiated ART, irrespective of treatment guidelines or antiretroviral regimens. The number of people initiating ART was provided by the HCDCP. The proportion of individuals initiating ART among the AMACS participants was also estimated and compared to that reported from the HCDCP.

#### Stage 5: Proportion retained in care

Proportion retained in care was estimated using AMACS data. Among all AMACS participants who had ever initiated ART, those with at least one clinic visit, reported being on treatment or with a laboratory test taken between 15/07/2012 and 31/12/2013, were considered as being retained in care. 95% CI of the estimated proportion was based on binomial distribution. Estimates of the absolute number of individuals retained in care, overall and by risk group, were adjusted for AMACS coverage (i.e. proportion of PLHIV on care participating in AMACS).

#### Stage 6: Proportion virally suppressed

The proportion of AMACS participants with an HIV-RNA measurement ≤200 copies/mL or below current assay detection limit (i.e. <50 copies/mL) at their last visit between 15/07/12-31/12/13, were considered as virally suppressed [10]. Patients in care and on treatment but without a viral load measurement available between 15/07/2012 and 31/12/2013 were considered as adherent and thus suppressed. 95% CI of the estimated proportion was based on the binomial distribution. The proportion of people virally suppressed over all PLHIV (overall and by risk group) was also estimated. To account for the two sources of uncertainty (i.e., in estimating PLHIV and in estimating percentage virally suppressed), a Monte-Carlo 95% CI was constructed.

In the primary analysis, patients at each stage were considered as a subgroup of those participating in the previous stage of the CoC. To provide an estimate comparable to the usual 4-stage CoC, the proportion of virally suppressed individuals among those who ever initiated ART, regardless of whether they were retained in care, was also estimated. In this approach, three estimates, the minimum, the maximum and the midpoint between these two, were generated by either including those who were lost to follow-up (thus not retained in care) but considering them as not supressed or by excluding them from the analysis. It should be noted that the maximum estimate as defined above does not coincide with the estimate of the primary analysis as in the latter case we only considered AMACS participants who were in care by the end of 2013.

### Sensitivity analysis

Stages 5 and 6 were estimated based on AMACS data under the hypothesis that AMACS participants consist of a representative sample of the diagnosed population. As this assumption may not hold, a sensitivity analysis was carried out. Briefly, AMACS participants and HIV diagnosed people as reported to HCDCP were matched and compared by age, sex, transmission mode (MSM, heterosexuals, PWID, other), region of origin (Greek vs non-Greek) and CD4 counts at diagnosis. The probability of being included in the cohort given demographic characteristics was estimated; weights inversely proportional to the inclusion probability were generated. Details of the applied method have been recently published [14]. Stages 5 and 6 were then re-estimated using the weighted AMACS data and weighted estimates were compared to the unweighted ones. Statistical analyses were performed using Stata software, version 14.0.

## Results

### Overall six-stage CoC

By the end of 2013, 13,627 individuals had been diagnosed with HIV and reported to HCDCP and 11,096 were alive; 7,657 of those alive were linked to one of the 13 collaborating clinics which were part of AMACS (overall coverage: 69.0%). The estimated number of PLHIV was 14,147 (95% CI: 13,691–14,588), corresponding to an HIV prevalence of 0.129% (95% CI: 0.124–0.133). Of these, 41.2% were MSM, 10.8% were PWID, 22.4% were heterosexuals, and for 25.6% the route of transmission was unknown.

Based on HCDCP data, the number of those linked to care was 9,544 (86.0%), an estimate close to that from AMACS data, when accounting for the number of HIV-infected individuals followed in clinics not participating in AMACS (9,257). Table 1 shows the number of people in the remaining stages of the CoC.

**Table 1:** The six-point Cascade of care for all HIV-infected individuals, for the subgroups of MSM, PWID, heterosexual men and women ^†^, and for people originating from a country other than Greece. Individuals in each stage are considered as a subgroup of the previous stage.

	All	MSM	PWID	Heterosexuals	Migrants*
**PLHIV**	14,147	5,832	1,523	3,165	-
**[Ν (95% CI)]**	(13,691–14,588)	(5,521–6,104)	(1,433–1,621)	(2,959–3,464)	
**Diagnosed**	11,096	5,133	1,325	2,454	2,158
**Ν (%, 95% CI)]**	(78.4, 76.1–81.0)	(88.0, 84.1–93.0)	(87.0, 81.7–92.5)	(77.5, 70.8–82.9)	
**Linked to care N (%)**	9,544 (86.0)	4,988 (97.2)	1,070 (80.8)	2,291 (93.4)	1,702 (78.9)
**Ever treated N (%)**	7,488 (78.5)	4,147 (83.1)	699 (65.3)	1991 (86.9)	1,199 (70.4)
**Retained on care**	6,466	3,692	611	1,623	847
**[Ν (%, 95% CI)]**	(86.4-, 85.5–87.2)	(89.0, 87.8–90.0)	(87.4, 83.9–90.4)	(81.5, 79.5–83.3)	(70.6, 67.2–73.8)
**Virally suppressed**	5,635	3241	438	1,441	721
**[Ν (%, 95% CI)]**	(87.1, 85.9–88.4)	(87.8, 86.1–89.3)	(71.7, 66.2–76.8)	(88.8, 86.0–88.7)	(85.2, 80.0–90.0)
Virally suppressed**	42.6	59.1	31.3	48.9	35.1
**[% (95% CI)]**	(41.0–43.9)	(56.4–62.4)	(28.3–35.0)	(45.2–53.5)	(33.1–37.1) ***

^†^ MSM: Men who have sex with men; PWID: People who inject drugs

*The number of migrants who were unaware of their status was not estimated

** Among PLHIV (persons virally suppressed who had not been treated were included)

*** Among the diagnosed

**Fig 1:**
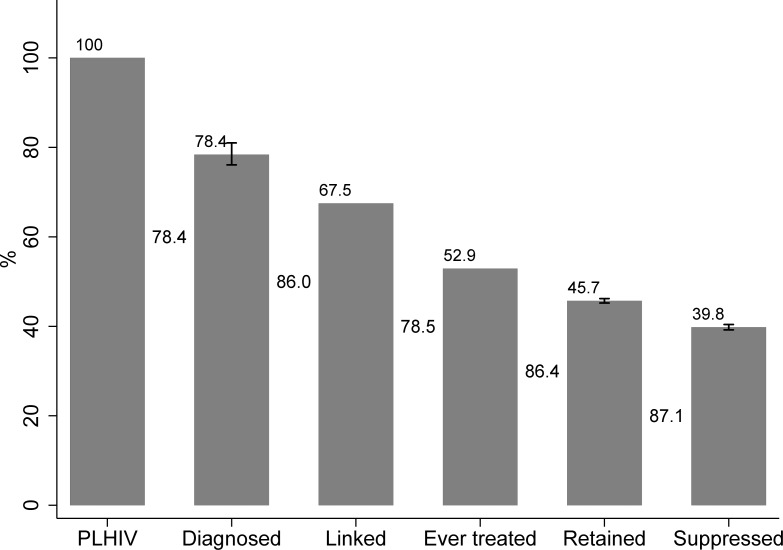
Cascade of care for the total population of PLHIV. The numbers between the bars correspond to proportion of the previous stage, while the numbers above the bars correspond to proportions of PLHIV. Vertical lines on the top of the Diagnosed, Retained to care and Virally suppressed bars correspond to 95% Confidence Intervals.

**Fig 2:**
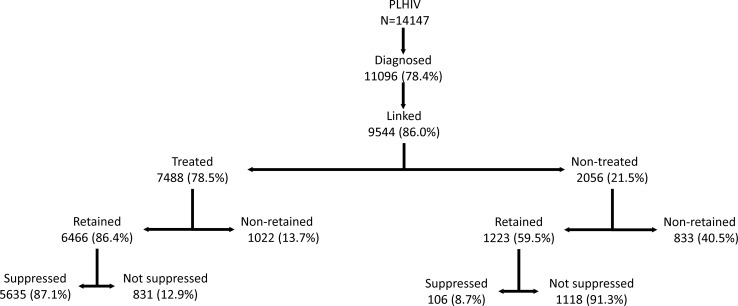
Flowchart of HIV infected individuals’ engagement in each stage of care, by the end of 2013. The estimates of the number of people who are retained to care, currently on treatment and virally suppressed are based on the AMACS cohort data.

The CoC for all HIV-infected individuals is presented graphically in Fig 1. Among the 14,147 individuals living with HIV, 78.4% (76.1 to 81.0) were diagnosed and 86.0% of them were linked to care. According to the HCDCP, 78.5% of the individuals linked to care were prescribed ART. The percentage of individuals initiating treatment in AMACS was similar (78.3%). Among treated individuals, 86.4% (95% CI 85.5–87.2) remained in care, and 87.1% (95% CI: 85.9–88.4) were virally suppressed. The corresponding numbers for the 4-stage CoC were: 78.4% of PLHIV were diagnosed; 67.5% of the diagnosed individuals had ever received treatment, and 81.2% of those ever treated were virally suppressed (min:75.3% max:87.2%). A detailed flowchart of all HIV-infected individuals is provided in Fig 2. Most of those who ever initiated ART were still on treatment at the end of 2013 (84.9%). Furthermore, these individuals were more likely to be retained in care than those who never began treatment (86.4% vs 59.7%, p<0.001; Fig 2). This result was observed in all subgroups but was more evident among heterosexuals (81.0% versus 37.6%, p<0.001). Most of the patients who remained in care, were virally suppressed at the end 2013 (87.1%; 95% CI: 85.9%-88.4%). Among treated individuals, 75.9% had viral load below 50 copies/ml and 82.7% had viral load below 1000 copies/ml at the end of 2013. An additional 1.1% of the linked-to care patients had viral load below 200 copies/ml, without ever receiving treatment (Fig 2). In total, of all PLHIV, 42.6% (95% CI: 42%-43.9%) were virally suppressed.

### CoC by risk group

**Fig 3:**
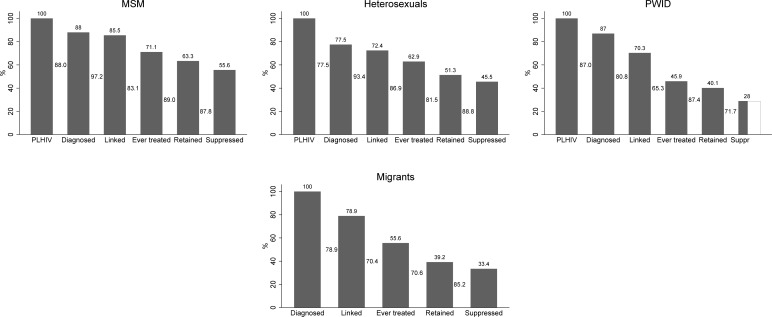
Cascade of care for men who have sex with men (MSM), people who inject drugs (PWID) and heterosexuals (A) and for migrants (B). The total number of migrants living with HIV could not be estimated; thus, the first stage of this CoC is the number of diagnosed migrants. The numbers between the bars correspond to proportion of the previous stage, while the numbers above the bars correspond to proportions of PLHIV for A and to proportions of diagnosed for B. Vertical lines on the top of the Diagnosed, Retained to care and Virally suppressed bars correspond to 95% Confidence Intervals.

The 6-stage CoC varied across HIV risk groups (Table 1 and Fig 3A). The percentage of diagnosed was lower among heterosexuals (77.5%) than among MSM (88%) or PWID (87%). A lower percentage of PWID was linked to care (80.8% of the diagnosed PWID, compared to 97.2% for MSM and 93.4% for heterosexuals). Only 65.3% of PWID linked to care were ever treated compared to 83.1% for MSM and 86.9% for heterosexuals. Nevertheless, PWID who initiated treatment, remained in care in similar percentages as the other groups of patients (Table 1). Based on a 4-stage CoC, the proportion of virally suppressed individuals among those who were ever treated ranged from 66.5% in PWID to 82.9% in MSM. Overall, including virologically suppressed individuals who were not treated, the percentage of viral suppression among PLHIV was 59.1% (95% CI: 56.4–62.4) for MSM, 31.3% (95% CI: 28.3–35.0) for PWID, and 48.9% (95% CI: 45.2–53.5) for heterosexuals.

The number of migrants living with HIV could not be accurately estimated because the overall number of migrants living in Greece and its secular trends were not available. Thus, the CoC for migrants was constructed starting from the second stage. In total, 2158 migrants were reported to be HIV-infected and alive by the end of 2013. Among those with known region of origin, almost half (47.5%) were originated from Europe (mainly from Eastern Europe), 40% from Africa, 9% from Asia, (mainly from South Asia) and 3.5% from South America, New Zealand and North America. These migrants are an established population in Greece, and it is very unlike that they have emigrated after their HIV diagnosis.

Among diagnosed migrants, approximately 79% were linked-to-care and of those 70.4% were ever treated. However, among those who initiated treatment, only 70.6% were retained in care at the end of 2013; thus, they had the lowest, rates of retention in care compared to all other groups (Table 1, Fig 3B). Although among migrants who were treated and in care, the percentage of those virally suppressed was similar to that in the other groups (85.2%; Table 1), among the diagnosed, only 756, i.e. 35.1%, (95% CI: 33.1%-37.1%) were virally suppressed at the end of 2013.

### Results from the sensitivity analysis

AMACS seems to capture a fairly representative sample of the diagnosed population in Greece as reported to HCDCP (S1 Table). Nevertheless, women, PWID, and people originating from another country were slightly under-represented in the cohort. The under-representation of these groups seems to be in line with their lower probability of being linked to care (Table 1 and S1 Table) indicating that AMACS, as expected, represents in fact those linked to care. To account for differential representation of subgroups of patients in the cohort, cohort’s participants were assigned inclusion weights. Weighted estimates for the percentage of viral suppression were similar to the unweighted ones, being 1% lower for PLHIV overall and 2% lower for heterosexuals.

## Discussion

### Main results

To our knowledge, this is the first effort to build a 6-stage CoC in Greece for all PLHIV, by risk group, and for migrants. At the end of 2013, there were weaknesses in the CoC in Greece, which also differed across risk groups. Overall, of PLHIV 21.6% were unaware of their status; 32.6% were not linked to care and 47% never initiated ART. Among those initiating ART, 86.4% retained in care and 81.2% were virally suppressed.

Our study revealed a high, but yet below the target, proportion of HIV-infected MSM who were diagnosed, but MSM have, in general, higher risk perception and willingness to access HIV testing services than other population groups [15, 16]. Interestingly, a high percentage of PWID with HIV were diagnosed. Until 2010, PWID represented a very low percentage of the total HIV-infected population. However, in 2011, an HIV outbreak occurred among PWID living in Athens [17]. As a response to the outbreak, Aristotle, a massive, seek-test-treat intervention was implemented [18]. It is estimated that more than 90% of PWID who lived in Athens between 2011 and 2013 were recruited and tested in Aristotle. It is thus likely that a high proportion of the HIV-infected PWID were diagnosed in the context of Aristotle and the intensification of harm reduction policies. The HIV epidemic among PWID is now largely contained but regular HIV testing, and harm reduction programs are and should be in place to avoid a new outbreak. The lowest percentage of diagnosed individuals was among heterosexuals (77.5%), probably reflecting the low-risk perception of this population.

In Greece, the total number of migrants living with HIV could not be estimated and the percentage of these who were diagnosed is unknown. Based on unpublished AMACS data, the percentage of those with late diagnosis (i.e., diagnosed with CD4<350 cells/mm^3^) is about 51% and it becomes 72% among those of African origin, rates similar to those reported in other European countries [19]. Barriers to HIV testing services, including structural barriers (e.g., language, cultural misunderstanding and legal issues), play a role in late diagnosis among migrants [20]. Expanding current HIV testing approaches may increase HIV testing uptake and yield [21]. In Greece, access to HIV self-testing is not available, while community-based testing, including lay-testing, remains limited in specific settings. Increasing access to HIV self-testing and community-based testing services and establishing channels to subsequent linkage to care may enable the identification of previously undiagnosed HIV cases.

In our analysis, we have demonstrated how surveillance data can be used to estimate the percentage of diagnosed people who are linked to care. As surveillance systems differ across countries, in the absence of relevant data, population-based HIV cohort studies can be used instead, especially when their representativeness have been accessed. In Greece, linkage to care is high among diagnosed MSM (97%) and heterosexuals (94%) but remains lower among PWID (81%) and among migrants (79%), highlighting of the challenge to developed tailored interventions to meet the needs of different subgroups of patients. Although ART is free of charge in Greece for all groups including undocumented migrants, a substantial percentage of HIV diagnosed individuals remained untreated. This is partly attributed to treatment guidelines which, until 2013, recommended that ART should be initiated only in patients with lower than 350 CD4 cell count/mm^3^. It is expected that numbers have improved following recent changes in treatment guidelines to align with WHO’s Treat-All guidelines [22]. According to unpublished HCDCP data (personal communication), at the end of 2016 there was a 15.5% increase in the proportion of HIV infected individuals receiving treatment compared to end of 2013, which indicates that the proportion of the diagnosed who started treatment has increased from 67.5% to 78%, corresponding to 90% of those linked to care. Given this increase, currently, an estimated 46% of PLHIV have achieved viral load suppression. Nevertheless, unless linkage-to-care is not prioritized within the existing HIV treatment services in Greece, it will not be feasible to achieve the target of 90% of those diagnosed to be on ART. The percentage of PWID and of migrants who initiated ART was shown to be low, even among those who were linked to care. This highlights the need for a multidisciplinary approach, including strategies to interconnect HIV clinics with addiction treatment centers and to provide HIV clinics with cultural mediators on a permanent rather than occasional base. The finding that those who initiated treatment, in all groups, are more likely to be retained in care, further supports the immediate treatment initiation.

The proportions of virally suppressed PWID and of migrants were lower than those in the other groups. This finding is in accordance with previous studies, which suggest that poverty, poor living conditions, and certain countries of origin are associated with lower likelihood of viral load suppression [23, 24]. Moreover, injecting drugs is associated with lower probability of being prescribed ART, being retained in care and, consequently, of achieving viral load suppression [25]. In our study, a relatively low, compared to the other risk groups, proportion of PWID that remained on treatment at the end of 2013 had achieved viral suppression, probably indicating intermittent non-compliance.

### Strengths and limitations

Greece has a well-organized HIV/AIDS registry and a nationwide HIV cohort with high geographical coverage. Proportions estimated using AMACS data could be biased if AMACS participants were not representative of the diagnosed population. In our sensitivity analysis we found that AMACS participants were representative of those linked to care rather than the diagnosed population. Therefore, proportions of virologically suppressed patients would be overestimated for the groups with lower rates of link to care (heterosexuals and migrants). However, the results after adjusting for under-representation of these groups showed that the degree of underestimation was in the range of 1 to 2%. In the main analysis, people lost-to-follow-up from cohort data (and thus not retained in care) were considered as non virollogically suppressed. However, some of those people may have been transferred to other HIV clinics. Ignoring this could lead to underestimated proportions of patients virologically suppressed. In AMACS, however, patients who transferred to another collaborating HIV clinic were tracked. Transfer to a non-AMACS site is possible, but given AMACS represented the largest clinics offering ART, the induced bias is expected to be very low. Another limitation of our study is that the percentage of people with HIV who had migrated was not available. Thus, the percentage of HIV-infected migrants who achieved viral suppression may have been underestimated. Nevertheless, migrants who have entered Greece by the end of 2013, mostly originated from Eastern Europe and Africa, had in fact migrated during early 1990’s and 2000’s and had become permanent residents of Greece. It should be noted that the migrants’ population in Greece, including those with HIV infection, up to the end of 2013, was rather stable and should not be confused with refugees’ waves in Greece after 2015, due to the war in Syria, which potentially comprise a moving population.

In this study, we characterized, overall and by HIV risk group, a 6 rather than the usual 4-stage CoC. We were able to determine the contribution of the additional stages (linkage to and retention in care) to the final target of achieving virally suppressed PLHIV and identify key gaps in the CoC for each HIV risk group [26]. Although we have now entered the “treat-all” era, and thus the ever treated stage is of less importance, linkage-to and retention-to-care remain very relevant [27]. In addition, all but linkage to care estimates were accompanied with their associated 95% CIs. For the estimates of the proportions of virally suppressed over all PLHIV, both sources of uncertainty (estimating PLHIV through ECDC tool and estimating percentage of virally suppressed from AMACS data) were considered. Estimates of the percentages of those linked to care and ever initiating ART were based on HCDCP data but were cross-checked in AMACS data. Here, we only provided a cross-sectional CoC. Time from seroconversion to diagnosis and from diagnosis to viral suppression and their secular trends could provide additional valuable information. However, this information is partially captured through our additional stages link-to-care and retention-to-care.

## Conclusions

By the end of 2013, Greece is not on track in achieving the UNAIDS 90-90-90 target. Barriers to achieving this have differed by risk group, with concerns over not under-diagnosing HIV across all populations, especially heterosexuals. Suboptimal linkage to care or reduced numbers of people who started treatment were the main issues among PWID and migrants. Interestingly, among those who initiated treatment, retention in care was substantially higher than among those who had never started treatment. These data provide useful information to policy makers. Of course, updated CoC are urgently needed to assess progression and the effects of the introduction of new treatment guidelines. The recent surveillance reports show that HIV diagnosis rates are barely decreasing (except for a post-outbreak decline in PWID), a finding that underlines the importance of timely diagnosis, and of engaging and retaining in care of PLHIV.

## Supporting information

S1 Table Characteristics of individuals diagnosed with HIV who were alive by the end of 2013 and were reported to HCDCP and to AMACS. Diagnosed individuals are presented by linkage-to-care status.
(DOCX)
