# Supplementary material for: HIV cascade of care in Greece: Useful insights from additional stages
Source: PLoS One. 2018 Nov 15;13(11):e0207355. doi: 10.1371/journal.pone.0207355 (PMC6237384; doi:10.1371/journal.pone.0207355)
Supplement: S1 Table — Diagnosed individuals are presented by linkage-to-care status. (DOCX) [file pone.0207355.s001.docx]

# Supporting Information

S1 Table. Characteristics of individuals diagnosed with HIV who were alive by the end of 2013 and were reported to HCDCP and to AMACS. Diagnosed individuals are presented by linkage-to-care status.

|  | **AMACS** | **HCDCP** | | |
| --- | --- | --- | --- | --- |
|  | **N=** **7657**  **N (%)** | **Linked**  **N=9544**  **N (%)** | **Not linked**  **N=1552**  **N (%)** | **All diagnosed**  **N=11096**  **N (%)** |
| **Sex** |  |  |  |  |
| Male | 6439 (84.1) | 7856 (82.3) | 1204 (77.6) | 9060 (81.7) |
| Female | 1218 (15.9) | 1682 (17.6) | 339 (21.8) | 2021 (18.2) |
| Unknown | 0 (0) | 6 (0.1) | 9 (0.6) | 15 (0.1) |
| **Transmission group**^†^ |  |  |  |  |
| MSM | 4091 (53.4) | 4988 (52.3) | 145 (9.3) | 5133 (46.3) |
| PWID | 723 (9.4) | 1070 (11.2) | 255 (16.4) | 1325 (11.9) |
| MSW | 1882 (24.6) | 2291 (24.0) | 163 (10.5) | 2454 (22.1) |
| Other | 87 (1.1) | 175 (1.8) | 13 (0.8) | 188 (1.7) |
| Unknown | 874 (11.4) | 1020 (10.7) | 976 (62.9) | 1996 (18.0) |
| **Origin** |  |  |  |  |
| Greece | 6330 (82.7) | 7427 (77.8) | 859 (55.3) | 8286 (74.7) |
| Other than Greece | 1036 (13.5) | 1702 (17.8) | 456 (29.4) | 2158 (19.5) |
| Unknown | 292 (3.8) | 415 (4.3) | 237 (15.3) | 652 (5.9) |
| **CD4 cell count** |  |  |  |  |
| <200 cells/mm3 | 2112 (27.6) | 1634 (17.1) |  | 1634 (14.7) |
| 200-350 cells/mm3 | 1595 (20.8) | 1236 (13.0) |  | 1236 (11.1) |
| 350-500 cells/mm3 | 1476 (19.3) | 961 (10.1) |  | 961 (8.7) |
| >500 cells/mm3 | 2385 (31.1) | 1507 (15.8) |  | 1507 (13.6) |
| Unknown | 89 (1.2) | 4206 (44.1) | 1552 (100) | 5758 (51.9) |
|  | **Median (IQR)** | **Median (IQR)** | **Median (IQR)** | **Median (IQR)** |
| **Age (years)** | 33.4 (27.9, 40.7) | 32.5 (22.5-37.5) | 32.5 (22.5-37.5) | 32.5 (22.5-37.5) |

† MSM: Men who have sex with men; PWID: People who inject drugs; MSW: Men/women who have sex with women/men
